# A comprehensive *in vitro* characterization of non-crosslinked, diverse tissue-derived collagen-based membranes intended for assisting bone regeneration

**DOI:** 10.1371/journal.pone.0298280

**Published:** 2024-07-15

**Authors:** Federico Barrino, Valentina Vassallo, Marcella Cammarota, Maria Lepore, Marianna Portaccio, Chiara Schiraldi, Annalisa La Gatta

**Affiliations:** Department of Experimental Medicine, Section of Biotechnology, University of Campania “Luigi Vanvitelli”, Napoli, Italy; Università degli Studi della Campania, ITALY

## Abstract

Collagen-based membranes are class III-medical devices widely used in dental surgical procedures to favour bone regeneration. Here, we aimed to provide biophysical and biochemical data on this type of devices to support their optimal use and design/manufacturing. To the purpose, four commercial, non-crosslinked collagen-based-membranes, obtained from various sources (equine tendon, pericardium or cortical bone tissues, and porcine skin), were characterized *in vitro*. The main chemical, biophysical and biochemical properties, that have significant clinical implications, were evaluated. Membranes showed similar chemical features. They greatly differed in morphology as well as in porosity and density and showed a diverse ranking in relation to these latter two parameters. Samples highly hydrated in physiological medium (swelling-ratio values in the 2.5–6.0 range) and, for some membranes, an anisotropic expansion during hydration was, for the first time, highlighted. Rheological analyses revealed great differences in deformability (150-1500kPa G’) also alerting about the marked variation in membrane mechanical behaviour upon hydration. Samples proved diverse sensitivity to collagenase, with the cortical-derived membrane showing the highest stability. Biological studies, using human-bone-derived cells, supported sample ability to allow cell proliferation and to prompt bone regeneration, while no relevant differences among membranes were recorded. Prediction of relative performance based on the findings was discussed. Overall, results represent a first wide panel of chemical/biophysical/biochemical data on collagen-based-membranes that 1) enhances our knowledge of these products, 2) aids their optimal use by providing clinicians with scientific basis for selecting products based on the specific clinical situation and 3) represents a valuable reference for optimizing their manufacturing.

## Introduction

Resorbable collagen-based membranes are widely used to promote guided tissue regeneration in the oral cavity [1–15]. Regenerative periodontal therapy, aiming at restoring the periodontal attachment apparatus impaired by periodontal disease, and the treatment of alveolar bone defects are the two main surgical dental procedures routinely supported by these devices [2]. The membranes are designed to be placed over the bone defect to physically exclude the rapidly proliferating epithelial and connective tissue cells, thus creating a protected compartment, where the slowly growing bone tissue can regenerate [2, 3].

A great variety of membranes are currently available to physicians and new products are continuously developed and launched. They are marketed as sheets of various sizes and mainly differ for 1) collagen type and source, with fibrillar type I and type III collagen from porcine or equine or bovine connective tissues (tendon, pericardium, skin, submucosa) as the most used; 2) the specific deantigenization process (thermal, enzymatic, based on the treatment with specific solvents), needed to achieve biocompatibility; and 3) the further chemical processing (i.e. crosslinking using formaldehyde, glutaraldehyde, diphenylphosphorylazide-DPPA; hexamethylenediisocyanate), if any, aimed to adjust the membrane resorption time and mechanical properties [4–16].

Besides being cell-occlusive to guarantee the barrier function, a membrane should meet other requirements, crucial for successful clinical outcome. It is required to be easily handled to ensure an ease and correct placement by the physician. Once implanted, it should withstand the compression of the overlying soft tissues without collapsing and exhibit suitable resorption profile, matching tissue repair. These latter features are key to preserve the membrane’s structural integrity and ability to maintain space separation as long as needed for the underlying bone tissue regeneration. In addition, membranes that can biochemically prompt bone regeneration would be highly attractive [3–11, 16–18]. All these aspects of the clinical performance are directly related to membrane features such as chemical composition, swelling, mechanical properties, porosity, thickness, surface morphological features, sensitivity to chemical and enzymatically-catalyzed degradation, specific interaction with cells from the host tissue. These features are, in turn, related to the type and source of collagen and to the overall processing up to the final marketed product.

On these grounds, availability of reliable biophysical/biochemical data on commercialized membranes would aid physician in the optimal use of available products and would represent a useful reference for developing new highly performing devices intended for the same application [8].

Characterization studies of this type of device have been intensifying [6, 9, 11, 13, 14, 19–23]. However, only the physico-chemical and/or resorption properties or the biological features of selected commercialized membranes have been mainly investigated [9, 13–16, 19–24]. Fewer *in vivo* studies investigating membranes-host interaction are available [5, 6, 11, 25, 26]. A complete characterizing profile of these products is still lacking. In this context, here we selected four commercially available non-crosslinked-collagen-membranes, produced from different collagen sources, and characterized them *in vitro* to assess the biophysical and biochemical features that are key for the performance *in vivo*.

The results were expected to provide a comprehensive panel of data for collagen-based membranes, potentially highlighting differences in behaviour, contributing to enhance our knowledge of these devices and to optimize their use, design and manufacture.

## Materials and method

### Materials

Four commercially available collagen-membranes, intended for the use in dental and orthopedic surgery, were tested. According to the information reported in the leaflets and or in the literature, they differ for the collagen source as reported in Table 1 [19, 20, 23]

**Table 1:** Composition and label of the tested materials.

Label	Composition
1	Type I collagen from equine tendon
2	Type I equine cortical and/or cancellous bone collagen
3	Collagen from equine pericardium
4	Collagen I and III bilayer membrane from porcine skin

Dulbecco’s Phosphate Buffered Saline (PBS) without calcium and magnesium was purchased from Corning, USA. Dulbecco’s Modified Eagle’s Medium (DMEM) and all cell’s reagents were obtained from Gibco (Thermofisher Scientific, Waltham, MA, USA). Collagenase Type I and dispase were provided by Gibco ref 17100–017. EtOH 99% (Fisher chemical). Tris-buffered saline (Sigma). Cell Counting Kit-8 (Dojindo EU GmbH). TRIzol® Reagent (Invitrogen, Milan, Italy), Reverse Transcription System Kit (Promega, Milan, Italy) and IQ™ SYBR® Green Supermix (Bio-Rad Laboratories, Milan, Italy). Radio-Immunoprecipitation Assay buffer (RIPA buffer 1x) (Cell Signaling Technology). Nitrocellulose membrane (GE, Amersham, UK), Ponceau Red (Sigma).

Primary rabbit polyclonal antibody anti-OCN (ab93876, Abcam, UK), mouse monoclonal antibody anti-Tubulin (sc-5286,Santa Cruz Biotechnology, USA), chemiluminescence suitable horseradish peroxidase-conjugated secondary antibodies (Santa Cruz Biotechnology, USA) and ECL system (Merck Millipore, Darmstadt Germany).

### Methods

#### Chemical and biophysical analyses

*a) FTIR-ATR Analysis*. FT-IR spectra were recorded using a PerkinElmer Spectrum One FT-IR spectrometer equipped with a MIR TGS detector. Spectral acquisitions were obtained by using the Universal Attenuated Total Reflectance Accessory (UATR) an excellent internal reflection accessory for the analysis of solids and liquids.

The technique is non-destructive and involves placing a sample on top of a crystal with a high refractive index. An infrared beam from the instrument is passed into the accessory and up into the diamond crystal. It is then reflected internally in the crystal, and back towards the detector. When the beam is reflected within the crystal, it penetrates the sample by a few microns; loss of beam penetration can be prevented using a pressure arm which allows good contact of the sample with the diamond crystal.

The background spectrum was collected from the diamond plate without samples. All spectra were obtained using 32 scans in the range from 4000 to 650 cm^−1^ with a 4 cm^−1^ spectral resolution. Each sample was analyzed in triplicate. The spectra were preliminarily analyzed using the application routines provided by the software package (“Spectrum” User Guide, PerkinElmer Inc., USA) controlling the whole data acquisition system.

*b) Morphological analysis*. Membranes were dried in a critical point dryer and sprayed with platinum-palladium (Denton Vacuum Desk V sputter coater). The Fe-SEM Supra 40 Zeiss scanning electron microscope (5KV, InLens detector) and Smart SEM Zeiss software were used for the observation. Both the surfaces of the samples as well as the section were observed

*c) Density and Porosity*. Membrane density was calculated as the membrane mass (*m*)/membrane volume (*V*_*t*_) ratio.

*m* was measured using an analytical balance (Mettler-Toledo, XS105 DualRange). The membrane volume V_t_ was calculated as membrane length × width × thickness. The length and the width for each membrane were the ones indicated by the manufacturers and checked using a ruler. Membrane thickness was measured using a rheometer (MCR 301, Anton Paar) allowing for the measurement of sample thickness while monitoring the force applied to the sample. A plate-plate measuring system was employed. After having set the zero gap, the sample was placed on the lower plate. The upper plate was moved towards the lower one until a 1.0–1.2N force was detected, indicating the contact with the membrane. The corresponding gap was taken as the value for the membrane thickness. For each membrane, the measurement was carried out at least in triplicate and the mean value was considered for the calculation of V_t_ (each single value differed from the mean value less than5%).

Membrane porosity was calculated as:

$$Porosity\;\%=\frac{V_{v}}{V_{t}}\cdot 100$$

where V_v_ = membrane void volume; V_t_ = membrane volume

*Vt* was calculated as reported above. The membrane void volume V_v_ was evaluated by means of the liquid displacement method as already reported with modifications [27–29]. Specifically, each membrane (dry membrane) was weighed (W_o_) and allowed to equilibrate in absolute ethanol. Then, the samples were withdrawn, lightly tapped on filter paper and weighed (W_e_). The void volume was calculated as:

$$V_{v}=\frac{W_{e}-W_{0}}{\rho e}\cdot$$

Where *ρe* was ethanol density.

Measurements were carried out at least in triplicate.

*d) Swelling*. Sample water up-take capacity was evaluated by gravimetric measurements using an analytical balance (Mettler-Toledo, XS105 DualRange). The whole membranes and/or portions equivalent to 1/3 of the membranes were used. Each sample was weighed (W_d_) and placed in a sterile container. 15.0mL of PBS were added to each sample and the suspensions were incubated at 37°C. After 24h of incubation, samples were withdrawn, blotted with filter paper to remove surface water and finally weighed (W_s_). The swelling ratio was calculated as follows:

$$Swelling\;ratio=\frac{W_{s}}{W_{d}}$$

where W_s_ = swollen sample weight; W_d_ = dry (initial) sample weight.

Experiments were run at least in triplicate and results were reported as the mean value ±SD.

Dimensional variation occurring with hydration was evaluated. Specifically, the length and the width of the dry sample and of the same after hydration were measured using a ruler. The thickness of the dry and hydrated sample was measured using a rheometer following the same procedure described in the previous paragraph (contact was considered to occur at force values in the range 0.2–0.4N). As for the swollen sample, attention was paid to maintain the sample fully hydrated during the measurement. Variation of each dimension was calculated as

$$membrane\;dimension\;(length\;or\;width\;or\;thickness)\;variation=\frac{dimension\;for\;the\;hydrated\;sample}{dimension\;for\;the\;dry\;sample}$$

Variation in sample area was also calculated as

$$membrane\;area\;variation=\frac{Area\;(\;length\times width)for\;the\;hydrate\;sample}{Area\;(\;length\times width)for\;the\;dry\;sample}$$

*e) Rheological characterization*. Rheological analyses were carried out using a MCR 301 (Anton Paar, Germany) rheometer equipped with a parallel plate geometry (25mm plate diameter) and a Peltier temperature control. Measurements were performed on dry membranes and on the same equilibrated in physiological medium, at 37°C. Strain sweep tests were carried out at a constant frequency equal to 1.59 Hz, over a strain amplitude in the range 0.001/0.01–100%. The G′ and tan delta values in the Linear Viscoelastic Range (LVR) were derived and reported. Mechanical spectra were recorded over a 0.159–10 Hz frequency range, at a strain value within LVR.

*f) Sensitivity to enzymatic degradation*. Membrane samples (5x5mm^2^), swollen at equilibrium in PBS, were weighed (W_0_) and placed in sterile containers. 2.0mL of collagenase solution (4U/mL) in PBS were added. Samples were incubated at 37°C. At diverse time intervals up to 78h, membrane samples were withdrawn and then blotted with filter paper to remove surface water and finally weighed (W_t_). Degradation was evaluated by monitoring mass decrease over incubation time. Specifically, the sample residual mass (%), at each time point, was calculated as:

$$Residual\;membrane\;mass\;\%=\frac{W_{t}}{W_{0}}\cdot 100$$

Degradation curves were obtained reporting, for each sample, the residual mass as a function of time. Experiments were performed at least in triplicate. Data were reported as the mean value ± SD.

### Biological evaluation

#### a) Cells

Following the protocols established by Jo et al. 2018 [30], with slight modifications, mesenchymal cells were isolated from human bone kindly provided by Department of Medical and Surgical Specialties and Dentistry, University of Campania “Luigi Vanvitelli”, Naples, (Italy). The isolation and use of primary human cells was formally approved by the Second University of Naples (University of Campania) Ethics Committee, approved on December 2005, Internal Registry: Experimentation n.914 and donors signed the University of Campania Internal Ethical Committee consent. Briefly, the bone, specifically, a femoral head, was washed with PBS (Dulbecco’s Phosphate Buffered Saline without calcium and magnesium, Corning, USA) and placed in a standard 60mm^2^-culture plate with an enzymatic digestion solution based on type I collagenase at 3 mg/mL and dispase at 4 mg/mL (Gibco, Thermofisher Scientific, Waltham, MA, USA). After 24 hours, the digested bone was removed and the isolated human primary cells were *in vitro* cultivated in DMEM (Dulbecco’s Modified Eagle’s Medium DMEM, Gibco Thermofisher Scientific, Waltham, MA, USA) supplemented with FBS (Fetal Bovine Serum, Thermofisher Scientific, Waltham, MA, USA) (10% v/v), penicillin-streptomycin (1% v/v), and Amphotericin B (1% v/v). The culture medium was changed every 48 hours and the cells maintained at 37°C in a humidified atmosphere with 5% (v/v) CO_2_. Cells were used at the 2^nd^ passage of *in vitro* culture. Considering that human bone-derived cells, obtained using similar protocols, have already been well characterized, a further characterization of the cell population obtained here was not considered crucial for the aim of the study [30–33].

#### b) Cell culture on membranes

The hydrated membranes were placed in a standard 24-well culture plate. 2.5×10^4^ cells aliquots were suspended in 10 μL of culture medium and seeded on biomaterials. Samples were incubated under cell culture conditions for 2 hours to allow cell attachment. Then, other 500 μL of medium were added. The cell-laden membranes were maintained at 37°C in a humidified atmosphere with 5% (v/v) CO_2_ for 7 days, replacing the culture medium every 48 hours. The same number of cells was seeded on cell culture plate and used as a control.

#### c) Cell viability

Primary cells isolated and seeded on membranes were grown until 7 days and, at diverse time intervals (24 h, 48 h and 7 days), their viability was assessed by Cell Counting Kit-8 (Dojindo EU GmbH) following the manufacturer’s protocol. The absorbance of the obtained solutions was measured at 450 nm using a Beckman DU 640 spectrometer (Beckman, Milano, Italy). Cell proliferation index at time *t* was calculated as following:

proliferation index (*t*) = A_450_ (t)/ A_450_ (*24h*)

#### d) qRT-PCR

After 7 days of *in vitro* culture on biomaterials, the primary cells were harvested and lysed with TRIzol® Reagent (Invitrogen, Milan, Italy) as previously described [34]. Then, the Reverse Transcription System Kit (Promega, Milan, Italy) was used in order to reversely transcribe 1 μg of total RNA into cDNA following the manufacturer’s instructions. Finally, a quantitative Real-Time PCR was performed by the IQ™ SYBR® Green Supermix (Bio-Rad Laboratories, Milan, Italy). Cells coming from two diverse patients were used for the seeding on the membranes and each culturing experiment was analyzed in duplicate therefore four values were obtained for the mRNA expression of osteocalcin (OCN), osteopontin (OPN) and bone sialoprotein (BSP). The mRNA expression values were normalized with respect to the glyceraldehyde-3-phosphate dehydrogenase (GAPDH) housekeeping gene. The specific primer sequences used for these analyses are reported in Table 2. The variations of each gene expression were calculated using the comparative threshold method (ΔΔCt = difference in ΔCt between the cells grown on biomaterials and control) and the results are reported as the normalized fold expression using the quantification of 2^-ΔΔCt^ method [35]. Analysis of relative gene expression was performed using the Bio-Rad iQ5 software (Bio-Rad, Milan, Laboratories).

**Table 2:** Primer sequences.

Gene	Forward primer	Reverse primer	AT PCR
***OCN***	5’-CTCCACATCCTCGCCCTATTG-3’	5’-CTTGGACACAAAGGCTGCAC-3’	58°C
***OPN***	5’-GCCGAGGTGATAAGTGTGGTT-3’	5’-GAGGTGATGTCCTCGTTCTG-3’	58°C
***BSP***	5’-CTGGCACAGGGTATACAGGGTAG-3’	5’-ACTGGTGCCGTTTATGCCTTG-3’	60°C
**GAPDH**	5′-TGCACCACCAACTGCTTAGC-3′	5′-GGCATGGACTGTGGTCATGAG-3′	55°C

#### e) Western blotting

To analyze the protein expression, in addition to the cells grown on membranes and on plate for 7 days, also cells grown on plate for 24 hours (time 0; t_0_) were used. In this regard, all the cells were harvested and lysed by a Radio-Immunoprecipitation Assay buffer (RIPA buffer 1x) (Cell Signaling Technology). Bradford method was used to determinate the intracellular protein concentration for each sample [36]. Western blotting analyses were performed as previously described [37]. Briefly, 10 μg of proteins were electrophoretically resolved on 12% SDS-PAGE, transferred to a nitrocellulose membrane (GE, Amersham, UK) (2 hours, 110 V) and equivalent loadings were verified by Ponceau Red (Sigma) staining. After that, the membrane was blocked using 5% skimmed milk Tris-buffered saline (Sigma) and 0.05% Tween-20 (TTBS) for 1 hour at room temperature. Primary antibody against OCN (Abcam, UK), (diluted 1:500) was incubated overnight at 4°C. After 24h, the membrane was washed through TTBS and chemiluminescence suitable horseradish peroxidase-conjugated secondary antibody (Santa Cruz Biotechnology, USA) (diluted 1:20000) was used to detect specific immunoreactive bands. An ECL system (Merck Millipore, Darmstadt Germany) was employed. Anti-Tubulin antibody (Santa Cruz Biotechnology, USA), diluted 1:1000, was used to normalize the protein levels of each analyzed biomarker. Finally, a semi-quantitative analysis of protein expression was performed by using the ImageJ program following the manufacturer’s protocol. Specifically, densitometric analysis was performed normalizing OCN protein expression with respect to TUBULIN expression for each sample.

#### Statistical analysis

All experiments were carried out at least in triplicate. Data were statistically evaluated by running a student t test for comparison of two data groups and One-way ANOVA tests followed by post hoc tests using Holm correction for multiple comparison. p values lower than 0.05 accounted for statistical significance.

## Results

### Chemical and biophysical characterization

#### a) FTIR-ATR analysis

**Fig 1:**
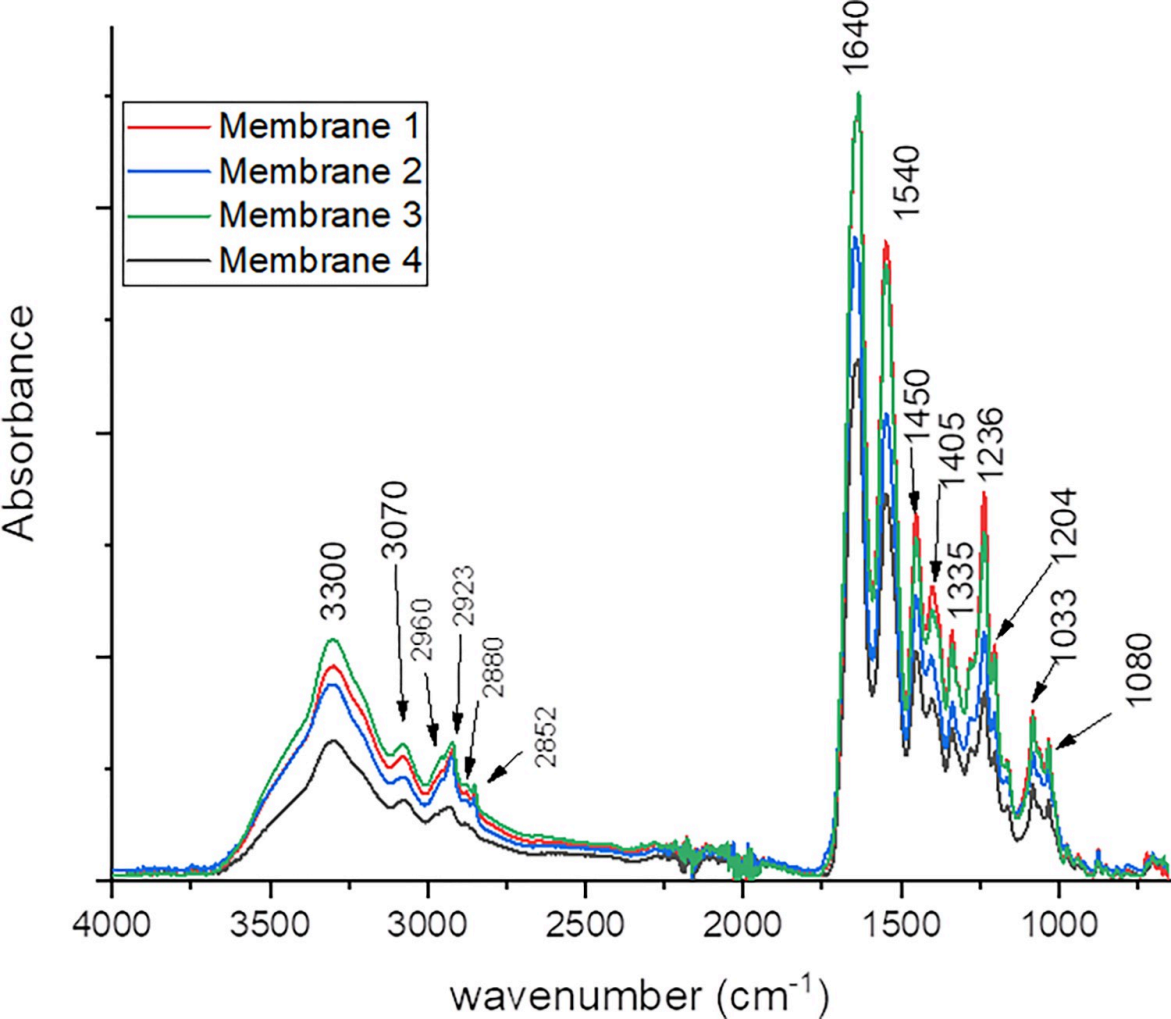
Fourier-Transform Infrared (FT-IR) spectra of membranes 1–4: Membrane 1 (red line), membrane 2 (blue line), membrane 3 (green line) and membrane 4 (black line).

The ATR FT-IR spectra of the membranes are reported in Fig 1. All spectra showed the characteristic collagen peaks with high absorbance in the regions 1500–1700 cm^−1^, moderate absorbance in the region 2800–3500 cm^−1^ and 1300–1500 cm^−1^ and relatively low average absorbance at 800–1200 cm^−1^. The possible absorption bands assignation is reported in Table 3 [38–40].

**Table 3:** Peaks values and assignments for the FT-IR spectra.

Peak value (cm^-1^)	description	Functional group
**3300**	Amide A	OH and NH stretching
**3077**	Amide B	= C-H and N-H stretching
**2960**	Methyl group	C-H asym stretching
**2923**	Methylene group	C-H asym stretching
**2880**	Methyl group	C-H sym stretching
**2852**	Methylene group	C-H sym stretching
**1634–1650**	Amide I	C = O stretching
**1545–1550**	Amide II	N-H bending and C-N stretching
**1450**		CH_2_ and CH_3_ bending
**1405**		
**1335**		CH_2_ and N-H bending; C-N stretching
**1283**	Amide III	N-H, C-N bending
		CH_2_ wagging
**1236**		
**1204**		
**1080**		C-O, C–O–C, C-N and C-OH stretching
**1033**		

#### b) Morphological analyses

**Fig 2:**
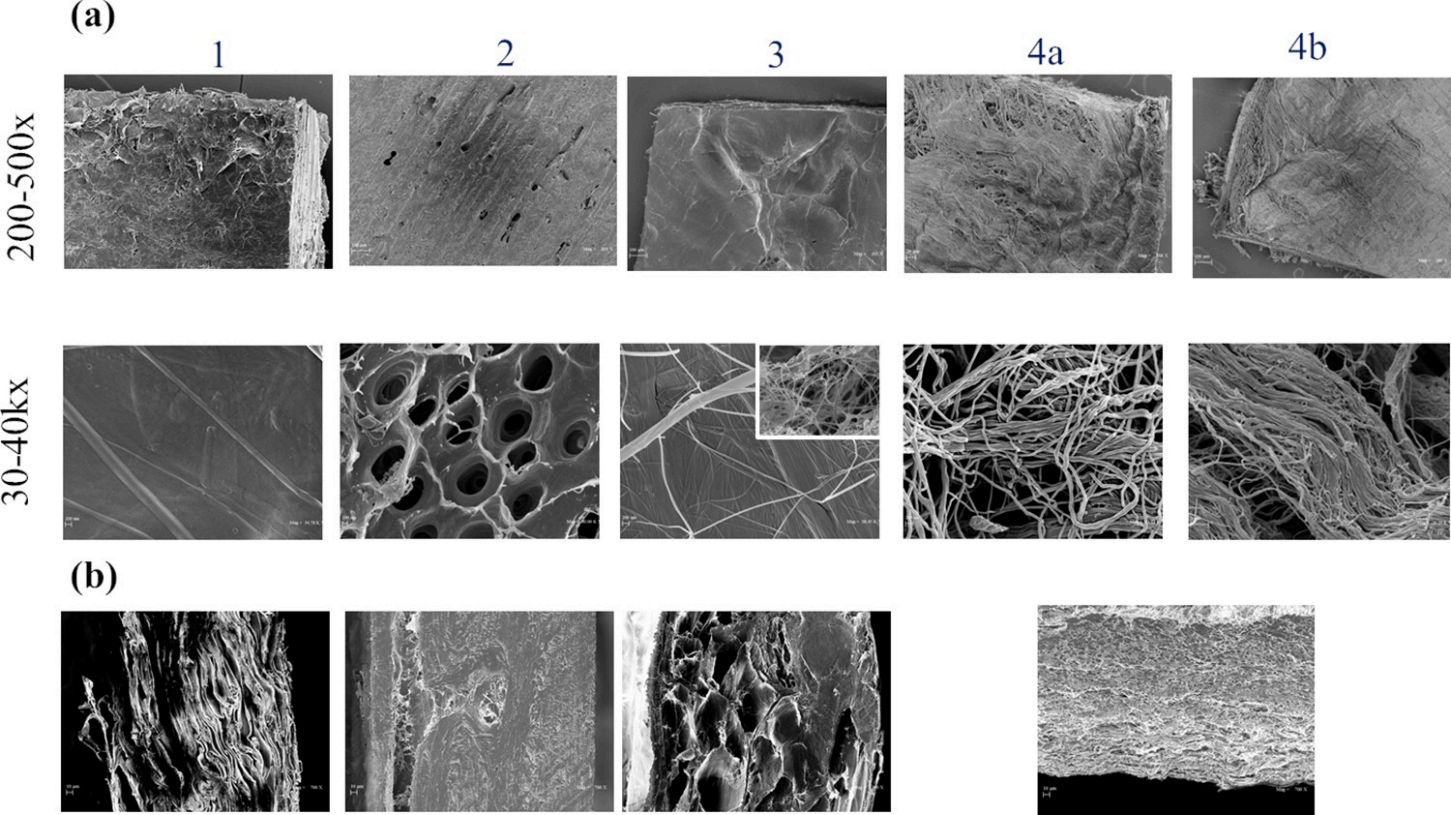
Representative SEM micrographs at different magnification of sample surface (a) and section (b) for membranes 1–4. For membrane 4, images for both the sides of the membrane (4a and 4b) are shown.

Representative SEM micrographs of the membranes, at different magnifications, are shown in Fig 2. Images of membrane surface and section are shown in Fig 2A and 2B, respectively.

As for the surface, at 200–500 magnification, membrane 1showed an irregular and rippled surface; membranes 2 and 3 showed compact and smoother surface with evident pores in the former; membrane 4 appeared more irregular, similarly to membrane 1 and presented both smooth and fibrous areas at both sides but the fibrous areas appeared denser on side a). Higher magnification (30–40 k×) highlighted further differences: 1) characteristic collagen fibrils with typical periodic banding pattern could be evidenced in all the membranes except membrane 2; 2) collagen fibrils were mostly tapered one over the other in sample 4, while in samples 1 and 2 they formed a more continuous, compact structure; however, areas showing well distinct fibrils were found also in sample 3, as shown in the frame. Pores in sample 2 were around 1μm in size. As for the section (Fig 2B), sample 1 exhibited an overlapping sheets structure. The section of sample 3 appeared porous with pores ranging from around 20 to around 70-80micron. Sample 4 exhibited two zones with the upper one similar to sample 3 but showing much smaller pores and the lower zone more resembling an overlapping sheets structure as found for sample 1. As well as for the surface, sample 2 was the most different in section morphology exhibiting the most compact structure.

#### c) Density and porosity

**Fig 3:**
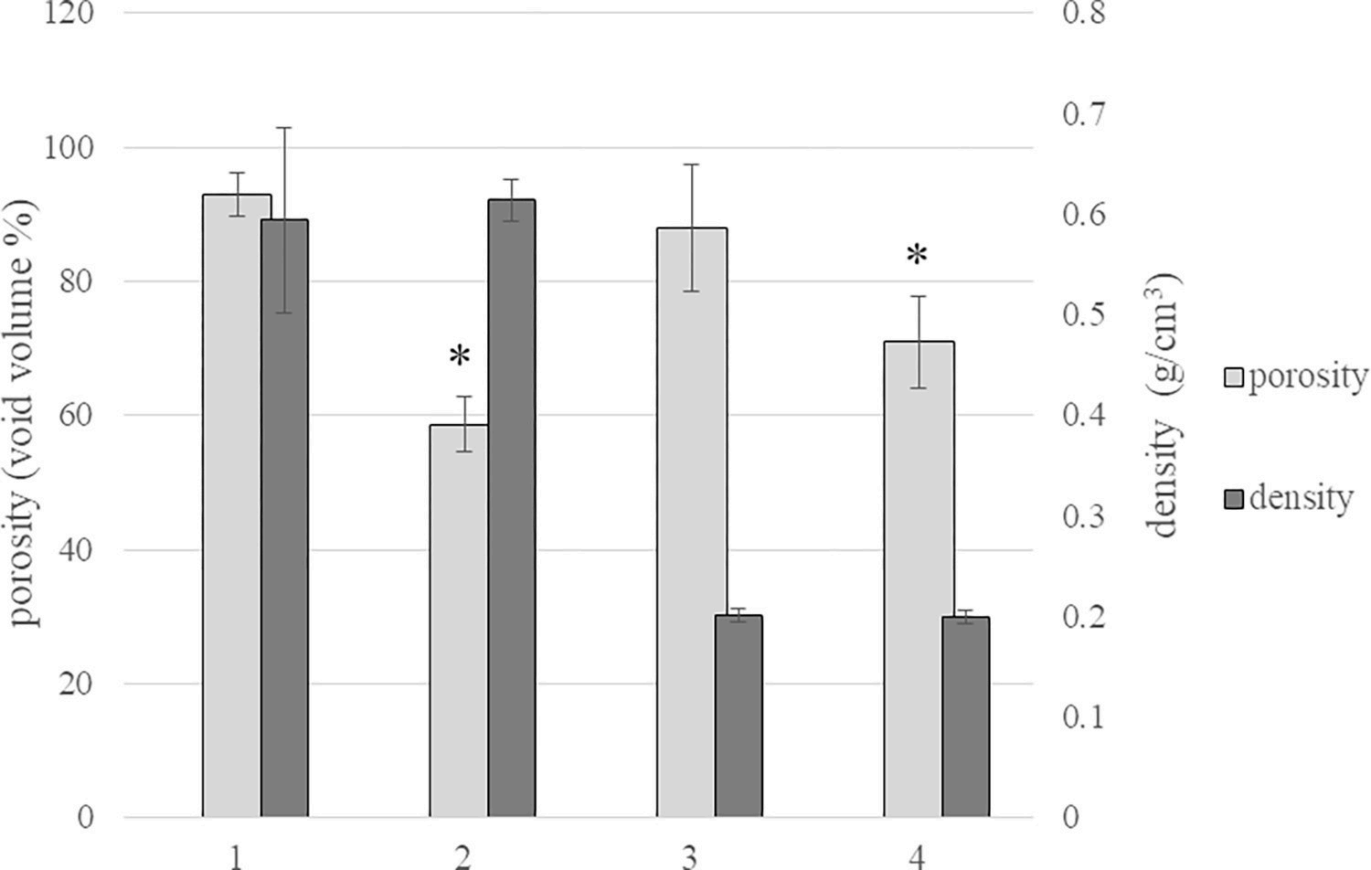
Density (g/cm^3^) and porosity (void volume/total volume %) data for the membranes 1–4. * p<0.01 *vs* other samples.

Density and porosity data are reported in Fig 3. Membranes 1 and 2 as well as membranes 3 and 4 were comparable in density with values around 0.6g/cm^3^ recorded for the former ones and around 0.2g/cm^3^ for the latter.

The highest porosity values (around 80–90%) were recorded for the equine tendon-derived collagen membrane (sample1) and the equine pericardium membrane (sample3) with no significant difference between them (p>0.05). The porcine dermis-derived collagen membrane (sample4) was very close in porosity with values around 70–80% while the equine cortical-bone derived membrane (sample2) was far less porous (59±4% porosity) than all the other samples.

#### d) Swelling behavior

**Fig 4:**
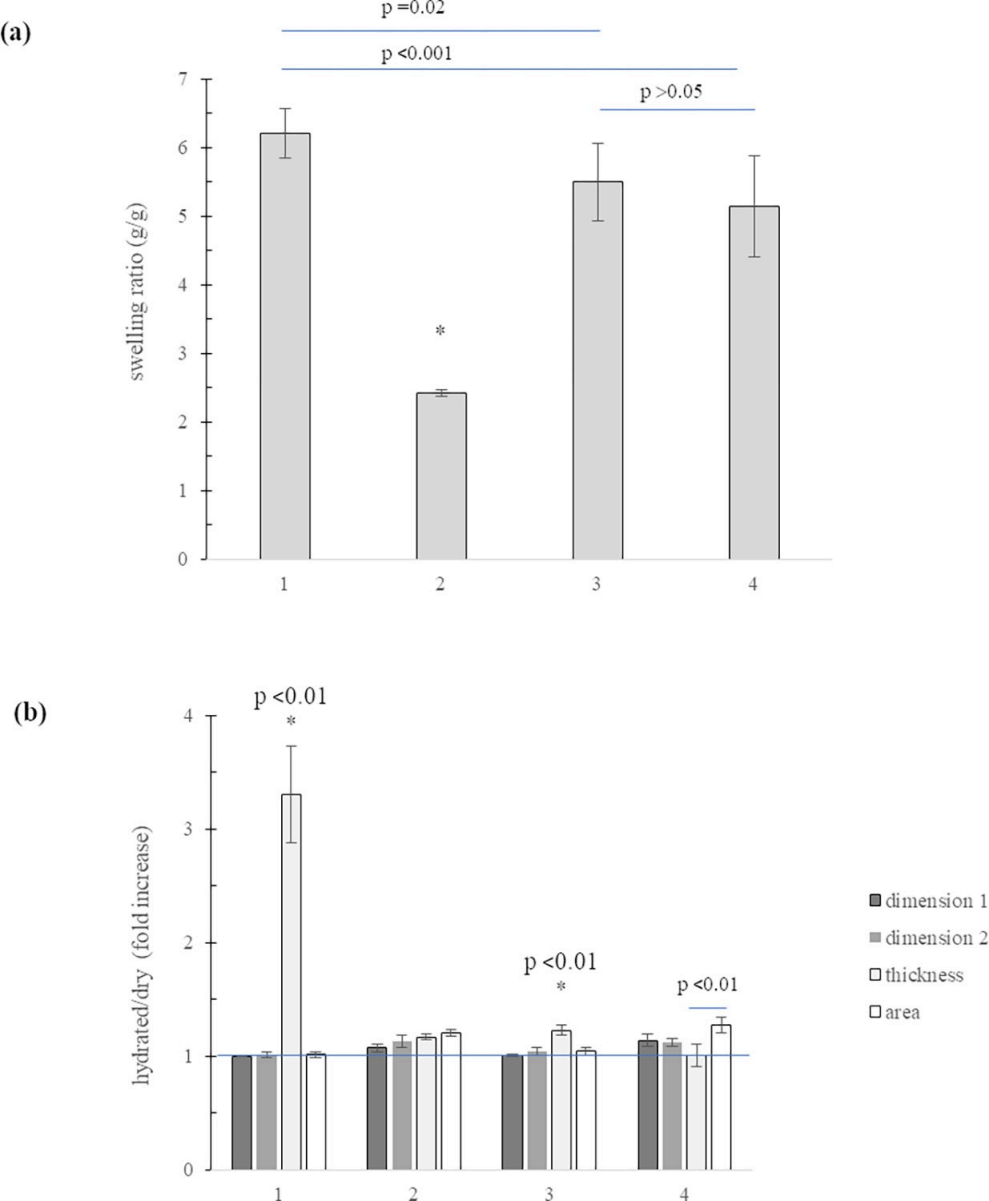
Membrane hydration behavior. (a) swelling ratio values at equilibrium in PBS at 37°C for membranes 1–4. * p<0.001 *vs* all the other samples (b) fold increase in membrane dimensions recorded after hydration in PBS. *p values *vs* the other dimensions. Variation in membrane area is also reported.

Data on membrane hydration behavior in PBS are reported in Fig 4. All the samples hydrated in physiological solution. The swelling ratio values at equilibrium are reported in Fig 4A. Samples increased their dry weight from about 2.5 to about 6-fold with the sample 1 showing the highest water up-take and sample 2 exhibiting the lowest water absorption value. Samples 3 and 4 showed intermediate and comparable behavior increasing their dry weight about 5–5.5-fold. Variation in membrane dimensions occurring due to hydration was reported in Fig 4B. Data demonstrated that samples 1 and 3 increased their size more in relation to thickness than in relation to the other dimensions thus highlighting, for these samples, an anisotropic behavior. The latter was slight for sample 3 and highly marked for sample 1. Samples 2 and 4 showed isotropic behavior with comparable enlargement in all directions (p>0.05). For samples 1 and 3, the membrane thickness increased more than the membrane surface area (p<0.01). As for sample 4, based on collected data, the size increase was comparable in relation to the three dimensions (p>0.05) but the area increase was significantly higher than the increase in thickness.

#### e) Mechanical properties

**Fig 5:**
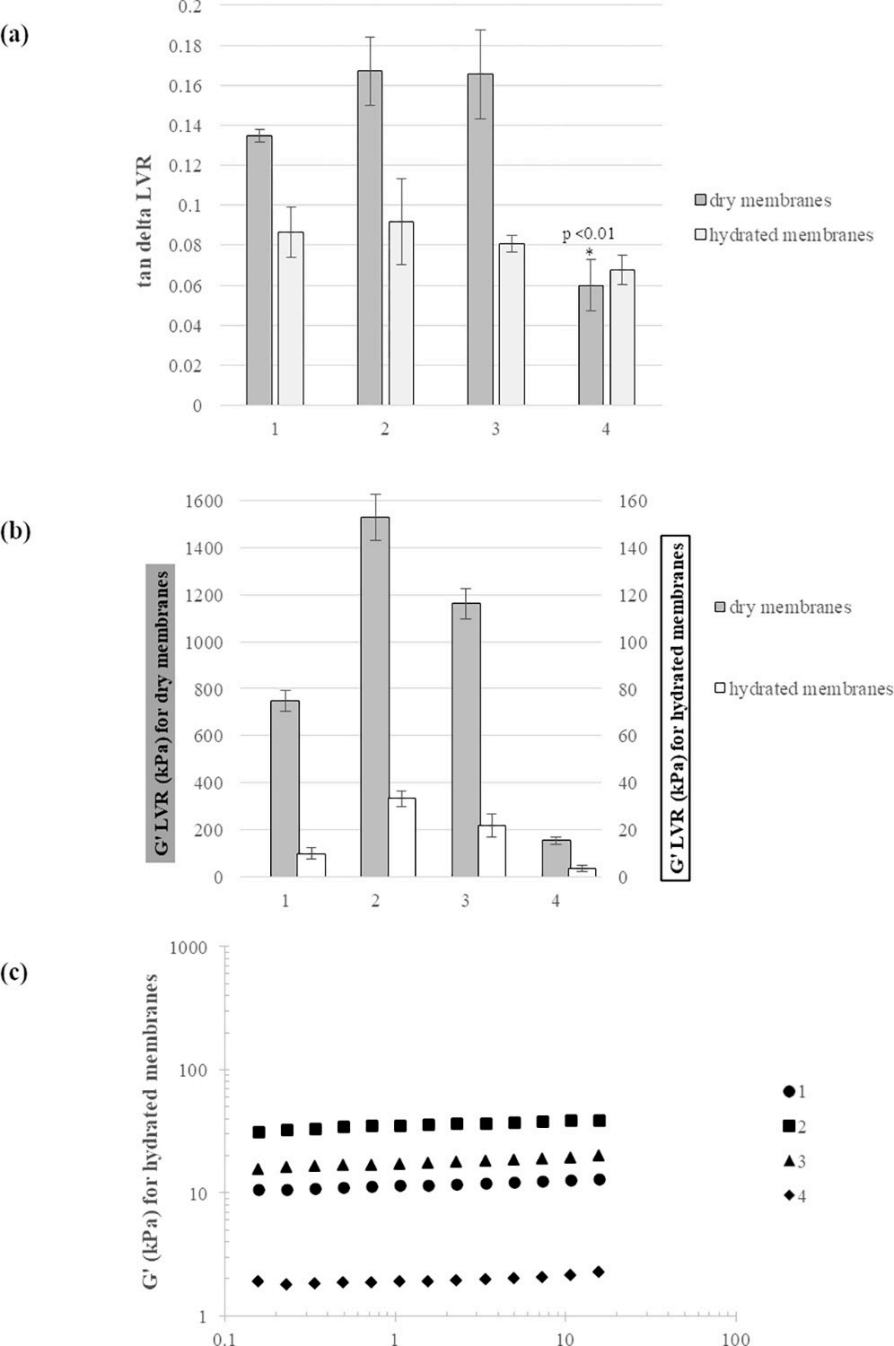
Membrane rheological behavior. Tan delta (a) and G’ (b) values, measured at 37°C, 1.59Hz constant frequency, within the linear viscoelastic range (LVR), for the membranes 1–4 in their dry state and at equilibrium in PBS. (c) G’ for membranes 1–4, in their hydrated state, as a function of frequency, measured at a constant strain (%) within the LVR, at 37°C.

Rheological analyses were carried out on membranes as commercialized and on the same after being equilibrated in PBS. Results are reported in Fig 5. Specifically, the tan delta and the Storage Modulus values, as measured within the linear viscoelastic range (LVR) at 1.59Hz constant frequency, are reported in Fig 5A and 5B, respectively.

As for tan delta, when compared in their dry state, membranes 1–3 behave similarly (tan delta values in the range 0.12–0.18; p<0.05) while sample 4 exhibited significantly higher overall elasticity (tan delta around 0.06; p<0.01). Upon hydration, no significant tan delta variation was recorded for sample 4 while, for samples 1–3, tan delta lowered to values comparable to that of membrane 4. The dry membranes highly differed for rigidity with G’ values ranging from 150 to 1500kPa. Specifically, sample 2 was the most rigid and sample 4 the most deformable sample. After hydration, a huge decrease in stiffness (around 40-70-fold decrease) was recorded for all the samples with membrane 1 varying more markedly. Sample relative rigidity was maintained with the stiffer and the most deformable samples (membranes 2 and 4, respectively) showing around 30kPa and 3kPa G’, respectively. The mechanical spectra of the samples, recorded at strain values within the LVR, indicated only a slight dependency of the Dynamic Moduli on frequency. The G’ values recorded as a function of the frequency, for the hydrated membranes, are reported in Fig 5C Comparable behavior was recorded for the moduli of the dry membranes.

#### (f) Sensitivity to enzymatic degradation

**Fig 6:**
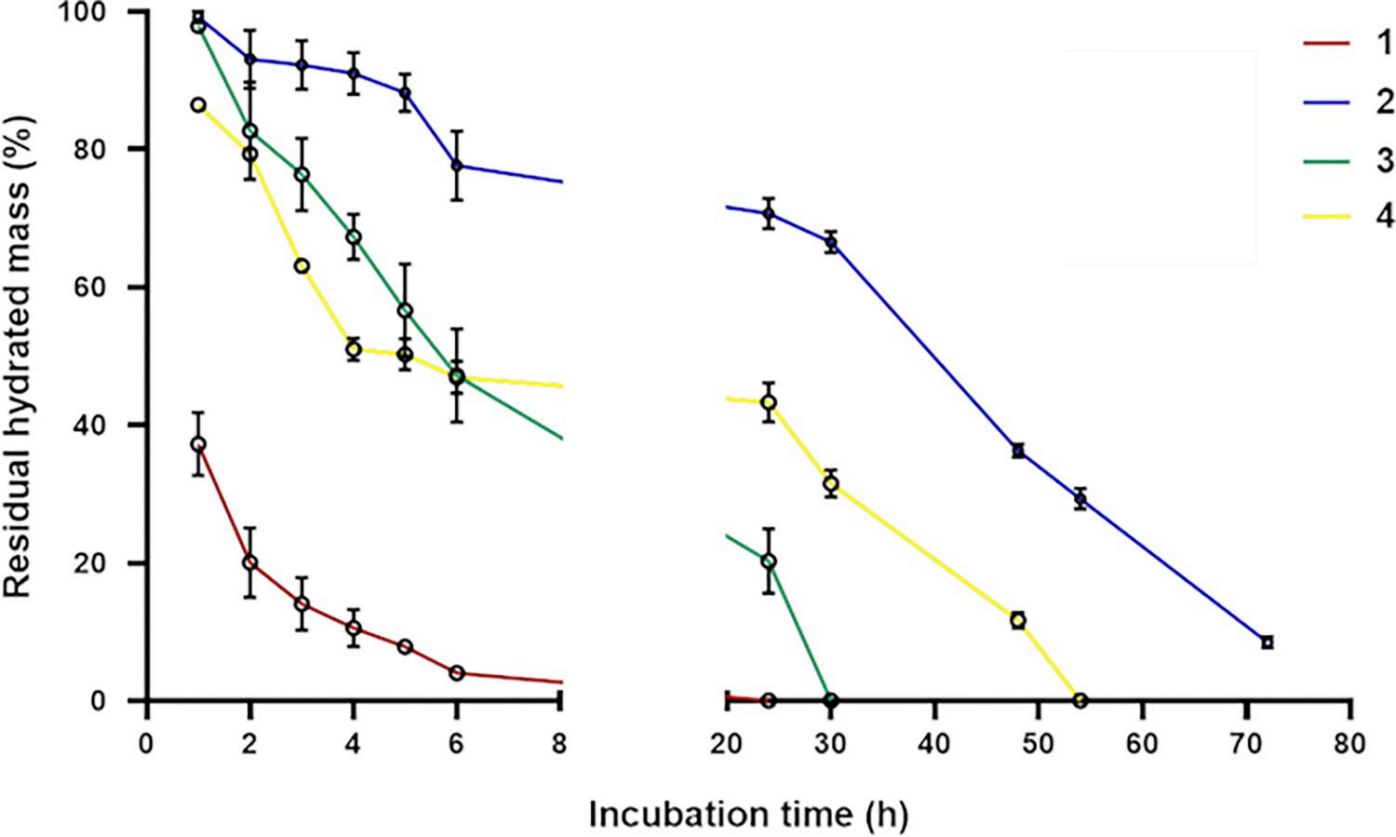
Membrane sensitivity to enzymatic degradation. Membrane residual mass (wt%) over time, during incubation with collagenase 4U/mL, pH 7.4, 37°C.

Curves showing sample degradation in the presence of 4U/mL collagenase are shown in Fig 6. Specifically, the residual mass for the samples (%) is reported as a function of the incubation time. Mass reduction was recorded for all the samples and differences in the degradation rate could be observed. Specifically, sample 1 degraded faster: at 3h of incubation, it reduced its weight by 85% while samples 2–4 retained about 63–93% of their initial mass. Complete degradation for sample 1 was recorded at 24h of incubation. Sample 2 showed the highest stability to enzymatic action with mass retention values significantly higher, compared to the other samples, over all the tested time interval. Samples 3 and 4 showed comparable stability within 6h (p = 0.002) while, at longer incubation time, sample 4 exhibited higher resistance. Data revealed that samples 2–4 showed gradual degradation within 5-6h. Sample 1 behave differently mainly degrading in the first hour of incubation and then more gradually, reducing its mass up to total solubilization. Similarly, membrane 4 lost 50% of its mass within 4 hours while the solubilization of the residual 50% mass needed around 30h.

### Biological evaluation

#### (a) Cell viability

**Fig 7:**
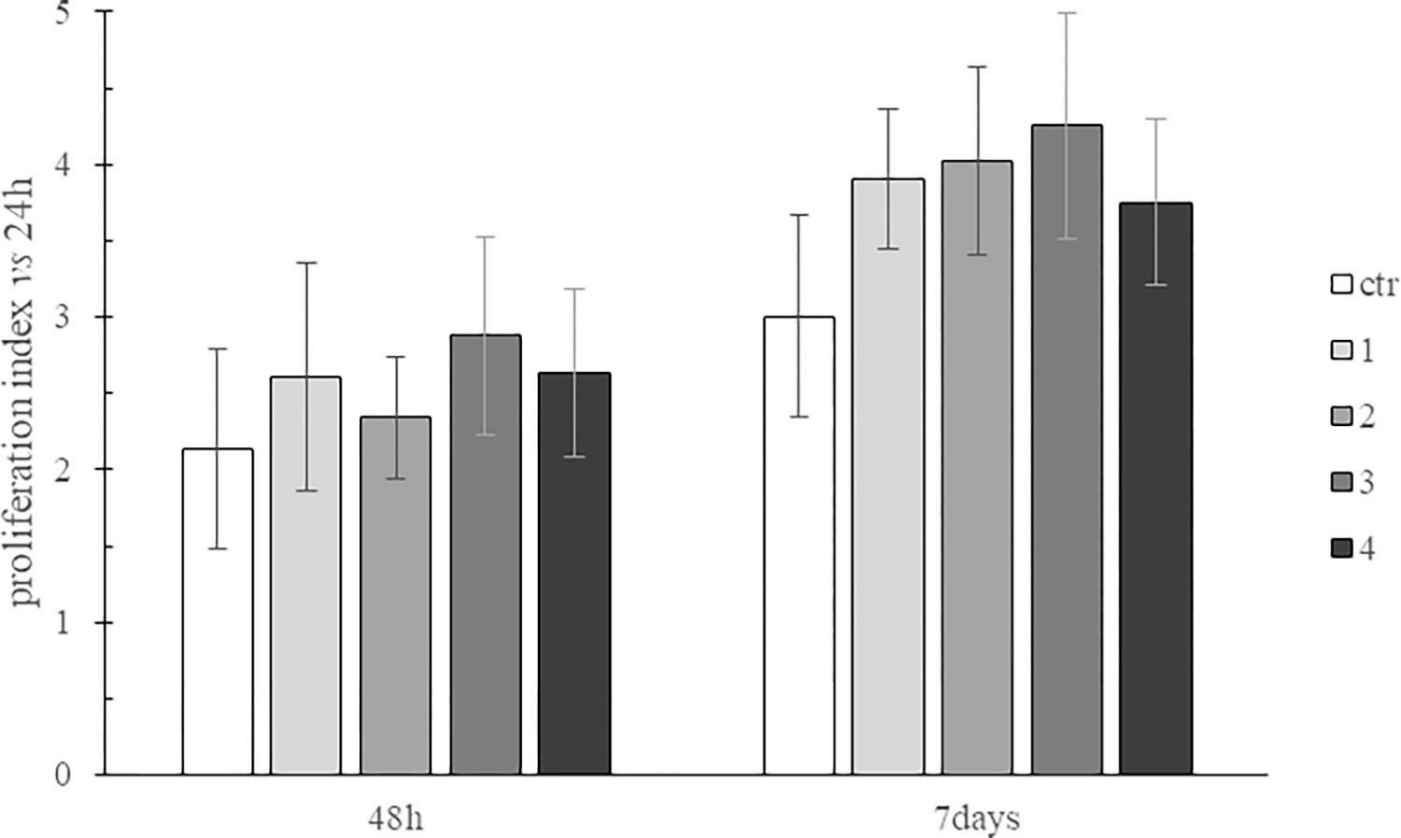
Proliferation index for primary human bone cells at 48h and 7days of culture on TCP and on membranes 1–4.

Primary bone human cells were seeded on membranes and on TCP (control; ctr). Cell viability was quantified over 7days of incubation and normalized to the values recorded at 24h to calculate the proliferation index. The cell proliferation index on the membranes and on TCP, as measured at 48h and 7days of incubation is reported in Fig 7. No cytotoxicity was observed. All the samples sustained cell viability and no significant difference was recorded neither among the membranes or when comparing the membranes to the control.

#### (b) qRT-PCR

**Fig 8:**
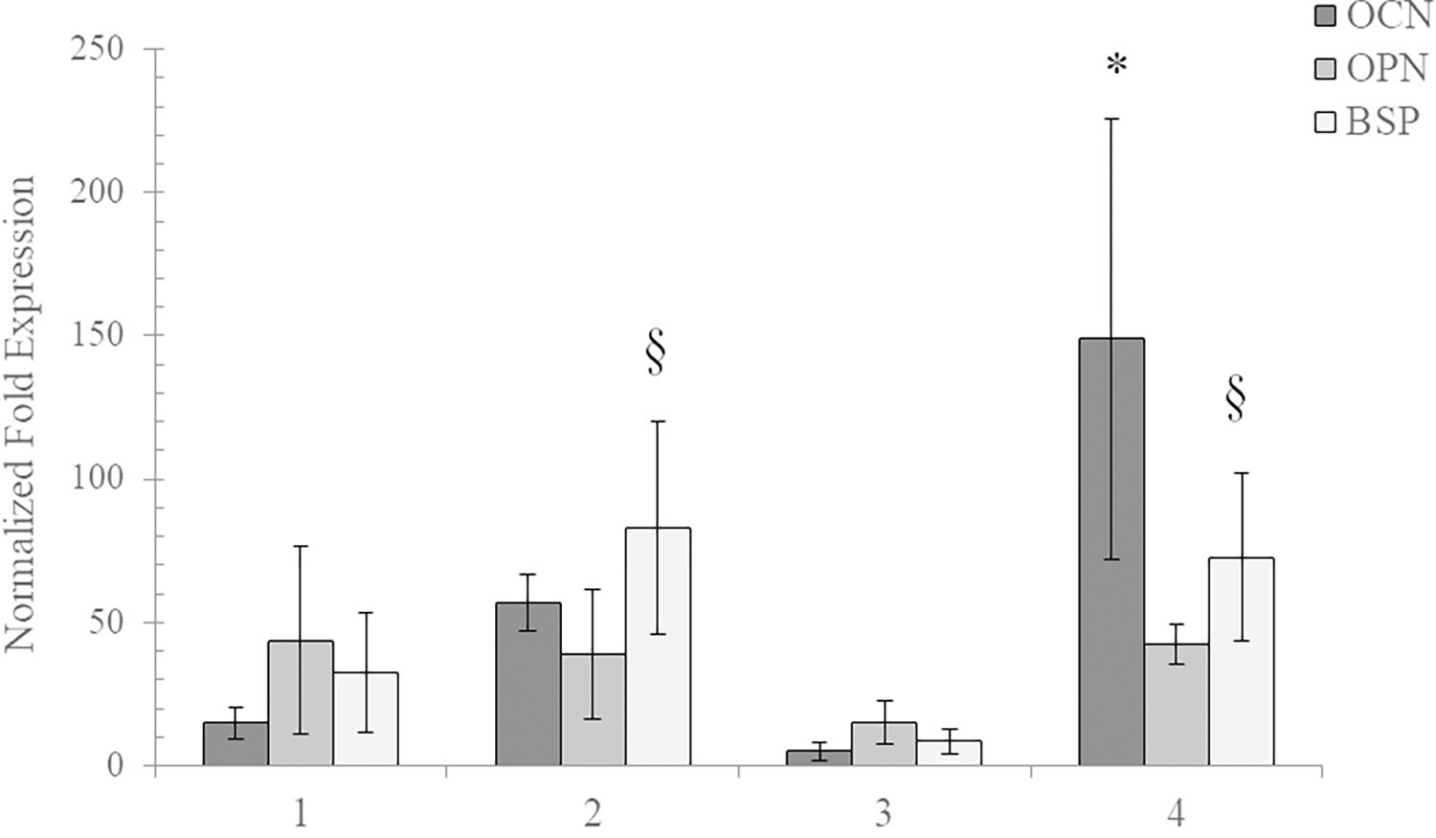
Gene expression analyses, after 7 days of *in vitro* culture, normalized to cells grown on the plate (ctr), for osteopontin (OPN), osteocalcin (OCN) and bone sialoprotein (BSP). The results are shown as mean ± S.D. * p<0.05 *vs* all the other samples; § p<0.05 *vs* sample 3.

Results of the gene expression analysis are reported in Fig 8. All membranes sustained the gene expression of three specific biomarkers (OPN, OCN and BSP) related to bone regeneration and phenotype. Specifically, all collagen-based samples enhanced the gene expression of bone tissue specific biomarkers in comparison to ctr (2^-ΔΔCt^ > 2 for all samples). OCN resulted more highly up-regulated in cells cultured on sample 4 compared to the other membranes (ANOVA; p<0.05). Moreover, BSP gene expression was significantly increased (ANOVA; p<0.05) in cells cultured on samples 2 and 4, compared to 3. Finally, a comparable up-regulation was found for OPN.

#### (c) Western blotting

**Fig 9:**
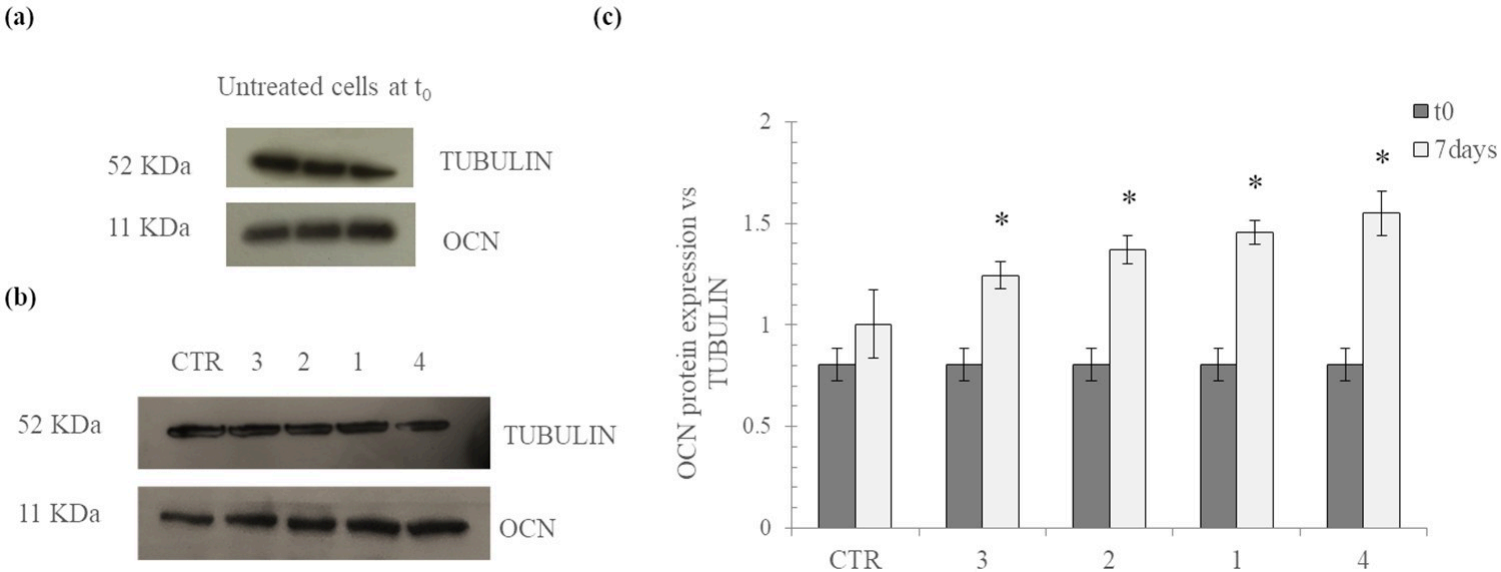
Evaluation of osteocalcin (OCN) protein level in bone isolated cells at t_0_ (a) and after *in vitro* culture of cells on the membranes and on TCP (ctr) for 7 days (b). Results of the densitometric analysis: OCN protein expression normalized to tubulin expression for cells at t_0_ and after 7days of culture on membranes 1–4 and on TCP (c). *p<0.05 *vs* t_0_.

The results of the western blotting analyses for the evaluation of the OCN expression are reported in Fig 9. In particular, the results for OCN protein level in bone isolated cells at t0 and after 7days of culture on TCP (ctr) and on membranes 1–4 are shown in Fig 9A and 9B, respectively. The results of the densitometric analysis are reported in Fig 9C.

As shown in the Fig 9A, at basal level (t_0_), the bone isolated cells expressed this marker. After the *in vitro* cultivation on the membranes for 7 days, OCN expression significantly increased with respect to t_0_ (p<0.05) regardless of the specific sample. This was not found for cells cultured on TCP (ctr): no significant increase in OCN expression after 7days compared to t_0_ was recorded_._ In addition, when comparing the fold increase in OCN expression at 7days *vs* t = 0, for cells cultured on the diverse membranes, all the samples proved comparably effective in prompting the protein expression level (fold increase values in the range 1.5–1.9), except for the sample 4 showing an OCN expression very slightly higher than sample 3 (ANOVA; p <0.05).

## Discussion

An extensive evaluation of commercial collagen-based membranes, comparing four devices from diverse tissues, in the same experimental *in vitro* set-up, for chemical, biophysical and biochemical properties key for the clinical performance is provided. Collected data represent a first comprehensive panel of data, valuable for the optimal use and design of these products.

Membrane chemical features, expected to affect tissue response and membrane resorption time, were comparable and typical of collagenous material. This suggests that the overall processing of collagen isolation and purification, up to the final products, does not leave appreciable contaminants. FT-IR data are also consistent with information from the labels since none of the membranes was reported to undergo post-purification chemical processing (i.e., crosslinking). Based on the above, potential differences in membranes performance should be attributed to other than the main chemical features.

SEM analyses highlighted considerable differences in membrane morphology, that are consistent with the diverse collagen sources. For collagen derived from soft tissues (membranes 1,2,4), characteristic collagen fibrils were evident with the structure of membrane 1 (section) resembling the typical parallel collagen fibril bundles of tendon tissue, while membrane 2 showed the typical morphological features of cortical bone [41–43]. As for surface morphology, likely affecting the *in vivo* response of cells to the device, the cortical derived sample was the most different. The observation of sample sections suggested diverse extent of porosity (membrane 2 as the least porous) and indicated diverse “morphology” for the void volume (void volume between compact layers for sample 1; round pores for membrane 3 and both types for membrane 4), possibly affecting other properties of the materials.

Membrane density was in the range reported in the literature for similar products [19, 20] and allowed us to identify a high-density group, formed by membranes 3 and 4 and a low-density group, encompassing membranes 1 and 2 and showing a mass-volume ratio around 3-fold lower. The authors underline how critical the measurement of membrane thickness is for density evaluation. Here, a method for measuring sample thickness, while avoiding its compression was employed for accurate evaluation. The latter does not apply to other reported measurements using an analogue caliper [13, 14, 44].

Density data refer to the mass-volume ratio of the membranes. The mass is the collagen-mass and the membrane total volume is the collagen- and the void-volume. Therefore, with equally dense collagenous material in the diverse samples, the higher the membrane density, the lower the void-volume (and, therefore, porosity) expected. However, the rank in membrane porosity did not resemble the one in density suggesting diversly dense polymeric material in the membranes. For instance, compared to membrane 2, the others have similar or higher density therefore comparable or lower porosity should be expected. However, membrane 2 showed the least porosity thus indicating the collagenous material forming this membrane has lower mass/volume ratio. These differences in collagen density can be related to the specific tissue features and overall processing of the natural material. The lower porosity value found for membrane 2 is in accordance with the more compact structure observed at SEM for this sample.

Membrane swelling behaviour was typical of hydrogels with values comparable to those reported for similar products [17]. The extent of sample hydration was consistent with porosity data: the more porous the membrane, the higher the hydration level in physiological medium. Like porosity, the swelling ability of the soft-tissues derived membranes was very similar and highly different from the bone-derived-sample.

To the best of our knowledge, the anisotropic size enlargement upon hydration, as found here for some samples, has not been reported elsewhere. The peculiar performance of sample 1 could be related to the morphological features of this membrane. Specifically, the filling by water of the void volume occurring between the compact collagen layers may be responsible for the markedly higher dimensional increase in thickness, compared to the one in the direction of the polymeric sheets. This aspect of membrane hydration behaviour is certainly key for a correct clinical use of these products.

Membrane mechanical properties are crucial for the ease of the *in situ* positioning and guard against collapse once implanted, thus affecting the efficiency of the barrier function. The rheological characterization confirmed, as expected considering the specific structural features of collagen, that all the samples behave as a covalently crosslinked network with Storage Modulus far exceeding the Loss Modulus and with a slight Moduli dependence on frequency.

Data revealed that the rigidity of commercial products spans over a very wide range. This alerts on how crucial is to provide physicians with mechanical data on membranes so that that they can select the most suitable for each specific defect. For instance, the most deformable material is expected to be easier to handle during the positioning *in situ* but less able to avoid collapse under a certain load and should be, therefore, better indicated for geometrically complex but small defects. On the contrary, more rigid membranes should better withstand high load after implantation.

Another key aspect of membrane mechanical performance was the drop in rigidity/increase in deformability accompanying hydration, as already reported for similar devices and also for other types of hydrogels [16, 19, 45]. Further, even if it is not the case for the specific comparison studied here, the rank in membrane rigidity could change after hydration, since the extent of G’ reduction depends on the specific collagen network. The same applies, as demonstrated by our data, for tan delta. Overall, considering that membranes are hydrated *in vivo*, the rheological data indicate that an accurate prediction of the (relative) sample mechanical performance requires evaluation after hydration. The higher deformability found for membrane 4 is in agreement with the greater flexibility reported in the literature for porcine-derived membranes [9].

Since enzymatically-catalysed hydrolytic degradation has been recognized as the main cause for premature membrane resorption, sample sensitivity to type I collagenase was evaluated [8, 9, 18, 46, 47]. Consistently with less hydration and porosity that are expected to reduce exposure to the enzyme, membrane 2 was the most resistant. However, the lower sensitivity of the bone-derived collagen of membrane 2 could also be related to the reported lower effectiveness of the Clostridium histolyticum type I collagenase at dissociating bone tissue [48]. Among the soft-tissue derived samples, showing comparable or very close porosity and hydration, membrane 1 was the less stable. The specific morphological features exposing large polymeric surface to the enzyme could be at least partially responsible for this finding. Beside the relative sensitivity to collagenase, the faster degradation in the early time interval found for sample 1 and the more gradual solubilization characterizing the other membranes should be also considered for optimal use. The relative stability to enzymatic action of membranes 1–3 is consistent with the data on the duration of the barrier effect reported in the product inserts.

Even if the main clinical effect claimed for these products is the physical barrier, it is evident and well-recognized that the final clinical outcome also depends on the biochemical interaction with adjacent cells [5]. Specifically, the more suitable for bone regeneration the environment created by the sample, the better and the faster the clinical outcome expected. Taking this into consideration, biochemical features were investigated. For this latter purpose, primary human cells isolated from bone were selected since they closely resemble the cellular environment interacting, *in vivo*, with the membrane, at the defect side. Bone-derived-cells are a heterogeneous cell population with proliferation ability and osteogenic potential [23, 49]. Membrane capacity to sustain bone-derived cell growth and differentiation into bone-phenotype was evaluated. For this latter purpose, OCN, OPN and BSP, playing key roles in bone formation and resorption were selected as the bone-phenotype biomarkers [49–52]. In agreement with other reports, our data indicated that bone-derived cells were able to attach to and proliferate on the diverse collagen membranes regardless of the specific source [12, 53]. This is key for the starting of the bone-tissue regeneration not only from the bony defect but also from the inner surface of the membrane.

There is controversy about collagen-membrane biological activity in prompting bone regeneration [3, 54]. Our gene expression data are in agreement with *in vivo* findings demonstrating an up-regulation of genes related to bone formation and remodelling in defects treated with collagen-membranes [3, 55]. In fact, at 7 days of *in vitro* culture on tested membranes, the bone-derived cells showed increased expression of all the bone-biomarkers tested, compared to control. When comparing the diverse membranes, considering that OCN marks terminal differentiation, being expressed within the final stages of the bone remodelling/regenerative process, [53, 54, 56, 57] while BSP should be considered involved in the main phases of the process enhancing the calcification process [57, 58], gene expression data may suggest membrane 4 as the one prompting faster regeneration, followed by membrane 2. OCN WB data confirmed membrane bioactivity towards bone regeneration but did not highlight great differences among the samples. Overall, based on the latter, the response of bone-derived cells to the tested membranes, recorded under our experimental conditions, suggests that the highlighted diverse biophysical behaviour of membranes does not translate into great diversity in biochemical potential.

## Conclusions

An *in vitro* chemical, biophysical and biochemical characterization of four commercial non-crosslinked collagen-based membranes from various sources was carried out.

The chemical and biophysical data highlighted comparable composition and demonstrated great differences mainly in membrane micro-structure, hydration, mechanical behaviour, and sensitivity to degradation. These findings allow us to predict diverse clinical performance for these products in relation to the handling and ease of positioning *in situ*, specific indication of use (based on the defect features and on membrane mechanical performance), ability to maintain the barrier function under certain load, and duration of the barrier action. An anisotropic expansion upon swelling was demonstrated for some membranes, with the tendon-derived sample showing the most marked anisotropy. This behaviour has been never reported before. The results of the mechanical characterization alert about the need for evaluating membranes after hydration for correct prediction of relative behaviour.

The study contributes to our understanding of the biochemical potential of these products. Regarding the ongoing controversy surrounding the collagen-membrane bioactivity, the biological and biochemical data collected here support sample ability to allow human bone-derived cell proliferation and to provide biochemical signalling towards bone regeneration. However, no relevant differences were recorded among the tested membranes, under our experimental conditions.

Overall, collected data increase our knowledge of these products, aid clinicians in selecting the most suitable product considering the specific clinical scenario and provide a useful reference for optimizing manufacturing towards specific performance.

## Supporting information

S1 File Original signals for western blotting analyses. (A) tubulin signals (on the left) and OCN signals (on the right) for cells grown on TCP for 24 hours (t 0) (B) tubulin signals (on the left) and OCN signals (on the right) for bone derived cells grown on TCP (CTR) or on membranes (sample 3, 2, 1, 4) for 7 days.
(PDF)

S2 File Minimal data set. (XLSX)
