# Supplementary figures and images for: A comprehensive in vitro characterization of non-crosslinked, diverse tissue-derived collagen-based membranes intended for assisting bone regeneration
Source: PLoS One. 2024 Jul 15;19(7):e0298280. doi: 10.1371/journal.pone.0298280 (PMC11249220; doi:10.1371/journal.pone.0298280)

(A)

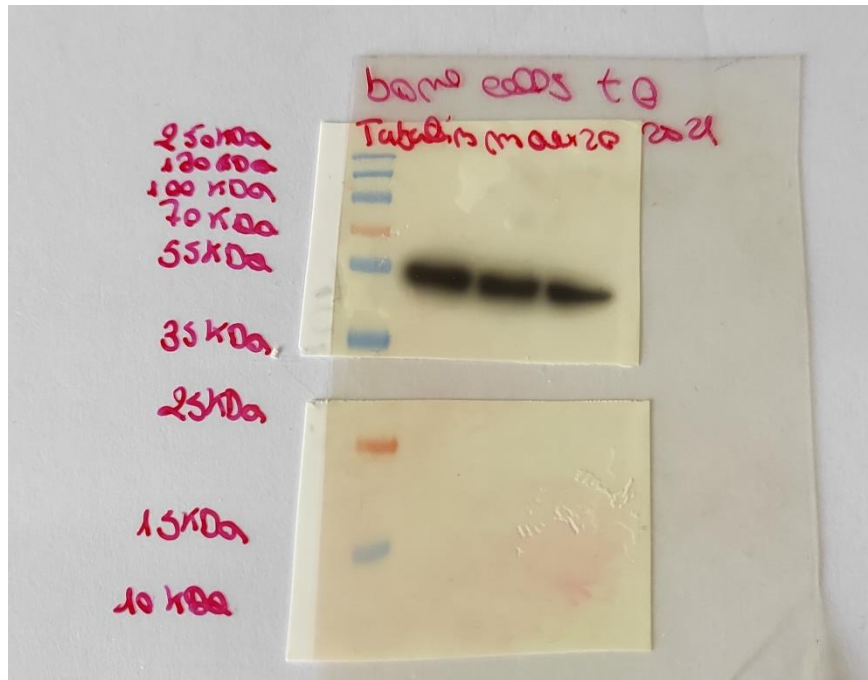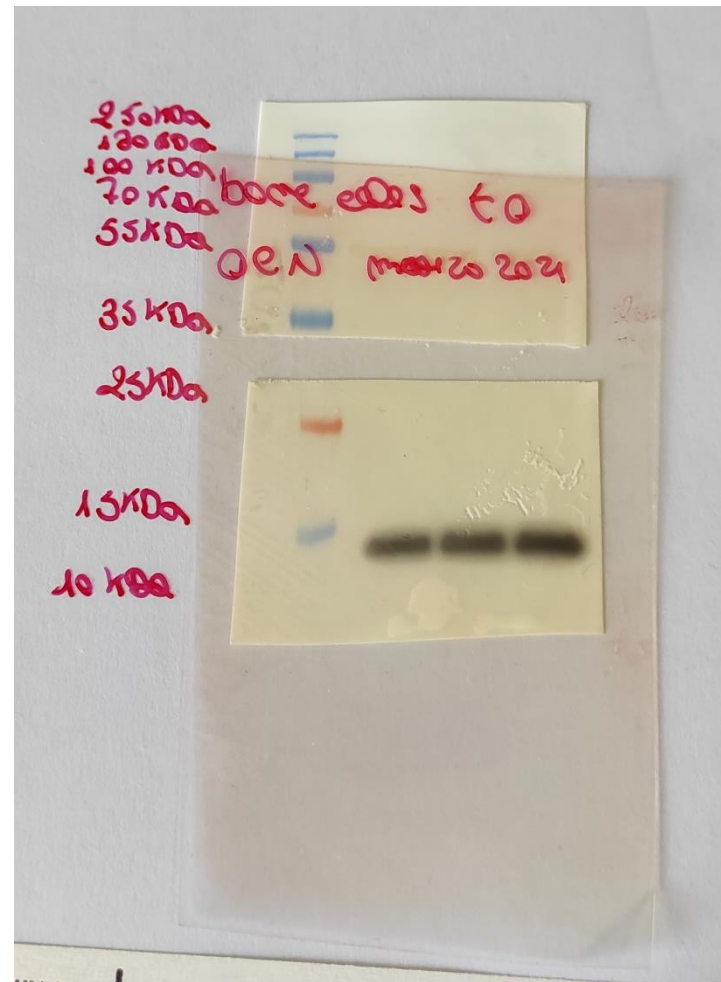

(B)

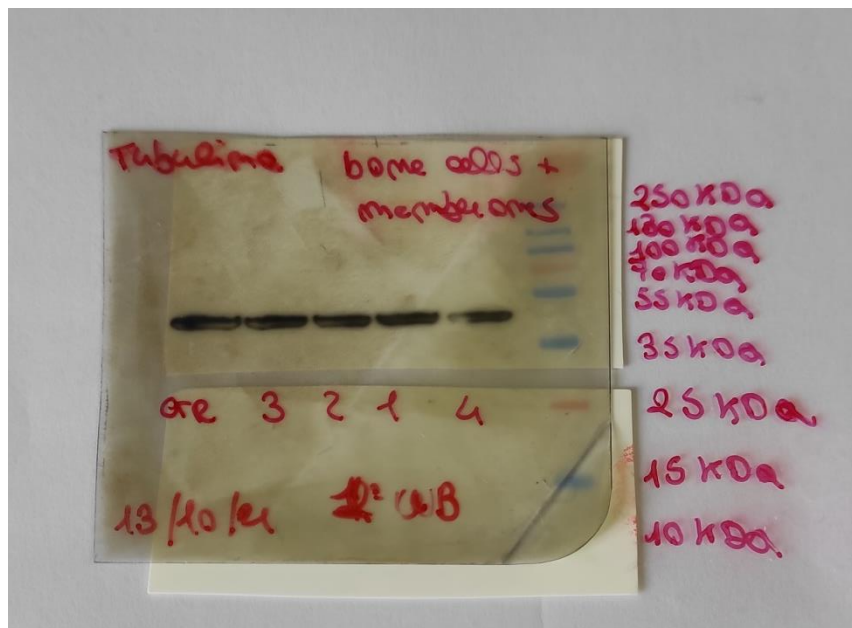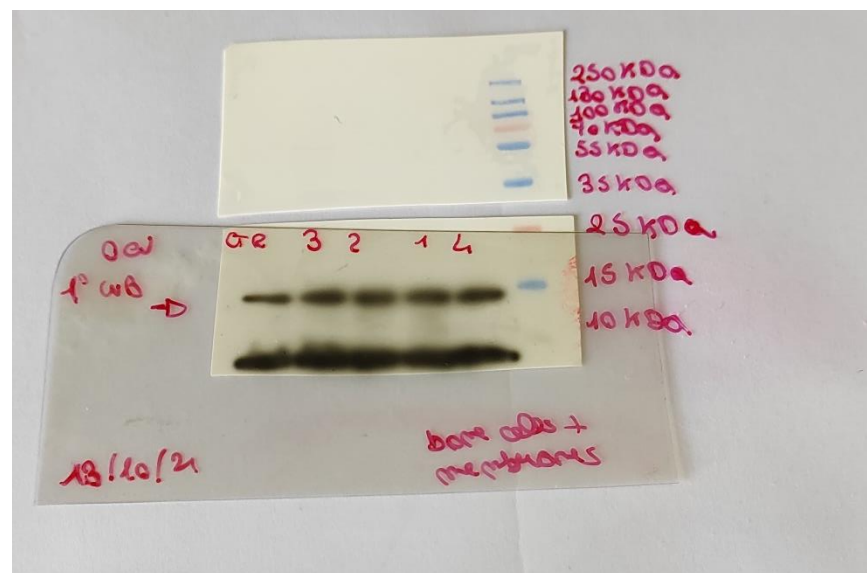

Supplement: S1 File — (A) tubulin signals (on the left) and OCN signals (on the right) for cells grown on TCP for 24 hours (t 0) (B) tubulin signals (on the left) and OCN signals (on the right) for bone derived cells grown on TCP (CTR) or on membranes (sample 3, 2, 1, 4) for 7 days. (PDF) [file pone.0298280.s001.pdf]
